# BH3 mimetic Obatoclax (GX15-070) mediates mitochondrial stress predominantly via MCL-1 inhibition and induces autophagy-dependent necroptosis in human oral cancer cells

**DOI:** 10.18632/oncotarget.11085

**Published:** 2016-08-05

**Authors:** Prasad Sulkshane, Tanuja Teni

**Affiliations:** ^1^ Advanced Centre for Treatment, Research and Education in Cancer (ACTREC), Tata Memorial Centre (TMC), Kharghar, Navi Mumbai-410210, Maharashtra, India

**Keywords:** MCL-1, Obatoclax, autophagy, necroptosis, mitochondria

## Abstract

We have previously reported overexpression of antiapoptotic MCL-1 protein in human oral cancers and its association with therapy resistance and poor prognosis, implying it to be a potential therapeutic target. Hence, we investigated the efficacy and mechanism of action of Obatoclax, a BH3 mimetic pan BCL-2 inhibitor in human oral cancer cell lines. All cell lines exhibited high sensitivity to Obatoclax with complete clonogenic inhibition at 200–400 nM concentration which correlated with their MCL-1 expression. Mechanistic insights revealed that Obatoclax induced a caspase-independent cell death primarily by induction of a defective autophagy. Suppression of autophagy by ATG5 downregulation significantly blocked Obatoclax-induced cell death. Further, Obatoclax induced interaction of p62 with key components of the necrosome RIP1K and RIP3K. Necrostatin-1 mediated inhibition of RIP1K significantly protected the cells from Obatoclax induced cell death. Moreover, Obatoclax caused extensive mitochondrial stress leading to their dysfunction. Interestingly, MCL-1 downregulation alone caused mitochondrial stress, highlighting its importance for mitochondrial homeostasis. We also demonstrated *in vivo* efficacy of Obatoclax against oral cancer xenografts and its synergism with ionizing radiation *in vitro*. Our studies thus suggest that Obatoclax induces autophagy-dependent necroptosis in oral cancer cells and holds a great promise in the improved management of oral cancer patients.

## INTRODUCTION

Oral squamous cell carcinoma (OSCC) is one of the most common cancers in Indian males and comprises 30–40% of all malignancies. Although it is primarily attributed to tobacco chewing habit, smoking and alcohol consumption [1], its association with Human Papilloma Virus (HPV) is recently emerging [2]. Indeed, a nationally representative survey revealed that, tobacco-related cancers account for about 42% male and 18% female cancer deaths across India [3]. About 40% OSCC patients die from loco-regional disease while 24% develop distant metastases [4]. However, despite major improvements in cancer therapeutics, the 5-year survival rate of OSCC patients has not shown a significant improvement over the past several years [5]. Hence, there is an urgent need to identify more effective therapeutic agents for the better management of OSCC.

p53 regulates the intrinsic pathway of apoptosis by modulating the expression and activities of BCL-2 (B cell lymphoma-2) family proteins [6–8]. The role of tobacco and HPV in inactivation of p53 is well documented [9, 10]. About 46% of oral cancer patients in India harbor inactivated p53 [10]. Apoptotic dysregulation is believed to be one of the key underlying reason for the progression of OSCC [11]. BCL-2 and BCL-XL overexpression correlates with therapy resistance and poor prognosis in OSCC [12–15]. We have previously reported overexpression of anti-apoptotic members of the BCL-2 family, particularly MCL-1 (Myeloid Cell Leukemia-1) over their pro-apoptotic counterparts in human oral cancers [16]. We also demonstrated predominant overexpression of antiapoptotic MCL-1 protein in oral cancer tissues versus normal and its association with therapy resistance and poor prognosis in oral cancer patients [16–19]. MCL-1 is a tightly regulated molecule, has a short half-life and is important for the development and survival of diverse cell types [20]. However, MCL-1 expression is frequently elevated in diverse human malignancies and is associated with therapy resistance. Several mechanisms including increased copy number, chromosomal and epigenetic changes and enhanced stability of MCL-1 protein contribute to its high expression in tumors [21]. Apart from its canonical prosurvival function, role of MCL-1 in mitochondrial homeostasis [22], DNA damage response [23, 24] and autophagy [25] are recently emerging.

All these studies suggests that the antiapoptotic proteins of the BCL-2 family, particularly MCL-1 are promising therapeutic targets in OSCC. Several pan-BCL-2 inhibitors are currently under development. However, therapeutic targeting of MCL-1 protein has largely been hindered by its structural discrepancy from other antiapoptotic BCL-2 family members [26]. But partial functional redundancy allows MCL-1 to substitute BCL-2, BCL-XL and BCL-W for their prosurvival function when they are inhibited or downregulated. MCL-1 is well known to exhibit resistance to ABT-737 and ABT-263 which potently antagonize BCL-2, BCL-XL and BCL-W [27–29]. Obatoclax (GX15-070), a BH3 mimetic is capable of inhibiting all the antiapoptotic proteins of the BCL-2 family, and potently inhibit the interaction between MCL-1 and BAK [26]. The potency of Obatoclax has been demonstrated against a variety of human cancer cell lines *in vitro* [26, 30–32] and in several clinical trials against diverse tumor types [33–35]. However, its activity against human oral cancers is rarely explored and largely unknown.

BH3-only proteins and BH3 mimetics are known to induce autophagy by activating multiple pathways [36, 37]. Autophagy has long been regarded as a cytoprotective mechanism deployed by tumor cells under stressful conditions [38]. However, sustained autophagy in response to a prolonged stress may lead to cell death when defective protein and organelle turnover exceeds the processing capacity of the cell [39]. A non-canonical pathway of cell death, Necroptosis has recently been shown to be linked to autophagy which involves a critical role of serine/threonine kinases called Receptor-interacting protein kinases (RIP1K and RIP3K) in a complex called Necrosome [40]. RIP3K further downstream recruits and phosphorylates its substrate Mixed Lineage Kinase Like (MLKL) which is proposed to execute necroptosis by mediating mitochondrial fission, generation of Reactive oxygen species (ROS) in mitochondria and recruitment of Ca^2+^ and Na^+^ ion channels or pore-forming complexes at the plasma membrane [41].

The present study demonstrates that Obatoclax mediates a caspase-independent, autophagy-dependent necroptosis in oral cancer cells associated with extensive mitochondrial stress. A late-stage block in autophagy leads to the association of p62 protein with RIP1K, RIP3K and FADD which triggers cell death by necroptosis. We also demonstrate the single agent *in vivo* efficacy of Obatoclax in xenograft mouse model. Additionally, we show the synergistic effect of Obatoclax with ionizing radiation treatment on oral cancer cells.

## RESULTS

### Obatoclax potently inhibits the clonogenicity of oral squamous carcinoma cells

**Figure 1:**
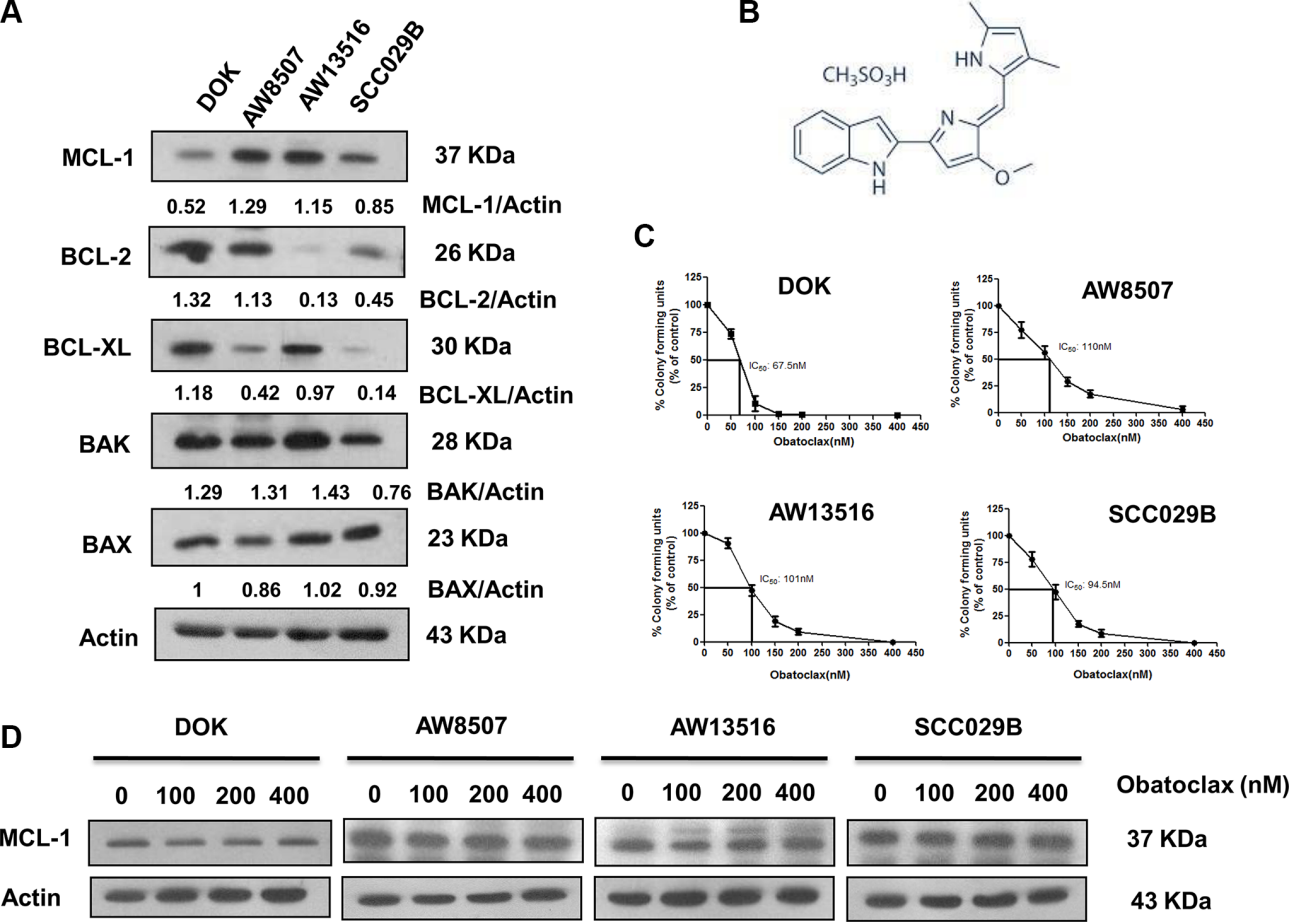
Obatoclax potently inhibits the clonogenic potential of oral cancer cells (**A**) Basal level expression of important pro and antiapoptotic BCL-2 family proteins in human oral cancer cells. β-actin served as loading control. (**B**) Chemical structure of Obatoclax. (**C**) Sensitivity of the four cell lines to Obatoclax was determined by the clonogenic assays. The survival (colony forming units) is expressed as percentage of vehicle controls. Data is represented as mean ± SEM of three independent experiments. (**D**) Effect of Obatoclax treatment on MCL-1 expression in the four OSCC cell lines. Images shown are representatives of triplicate experiments.

We demonstrated the efficacy of Obatoclax against four oral cancer cell lines (DOK, AW8507, AW13516, SCC029B). The basal levels of important pro and antiapoptotic BCL-2 family proteins were assessed by western blotting (Figure 1A). DOK expressed low levels of MCL-1 protein as compared to that of AW8507, AW13516 and SCC029B cell lines. Notably, all the cell lines expressed relatively higher levels of at least two of the three predominant antiapoptotic BCL-2 family proteins. We then performed the *in vitro* clonogenic assays. The plating efficiencies for all the four cell lines differed markedly (DOK: 30–40%, AW8507: 60–70%, AW13516: 70–80%, SCC029B: 55–60%). Obatoclax (Figure 1B) inhibited the clonogenic potential of these cells in a dose-dependent manner with complete growth inhibition at 200–400 nM concentration (Figure 1C). The sensitivities of the four cell lines to Obatoclax correlated significantly (*p* < 0.05, *R* = 0.96) with their MCL-1 expression which is in agreement with previous reports [32, 42]. DOK (IC_50_: 67.5 nM) exhibited highest sensitivity to Obatoclax with complete growth inhibition at about 100 nM concentration (correlates with its relatively lower MCL-1 expression) whereas AW8507 (IC_50_: 110 nM), AW13516 (IC_50_: 101 nM) and SCC029B (IC_50_: 94.5 nM) were relatively less sensitive possibly due to relatively higher MCL-1 expression. Obatoclax is shown to induce cell death in head and neck squamous carcinoma cells (HNSCC) by reducing MCL-1 expression [43]. We therefore assessed whether Obatoclax affects the expression of critical proteins of the BCL-2 family. Exposure of the four cell lines to Obatoclax for 24 hours revealed no significant alterations in the expression of either MCL-1 (Figure 1D) or other members of the BCL-2 family except for BIM and NOXA proteins, which showed a dose dependent reduction in expression (Supplementary Figure S1). Nevertheless, Obatoclax not only dissociated the constitutive interaction between MCL-1 and BAK in the mitochondrial outer membrane (Supplementary Figure S2A) but also induced BAX translocation to the mitochondria. Both these events are critical for Mitochondrial Outer Membrane Permeabilization (MOMP). However, we were not able to detect a significant cytochrome c release from the mitochondria to the cytosol (Supplementary Figure S2B).

### Obatoclax mediates caspase-independent cell death in OSCC cells

**Figure 2:**
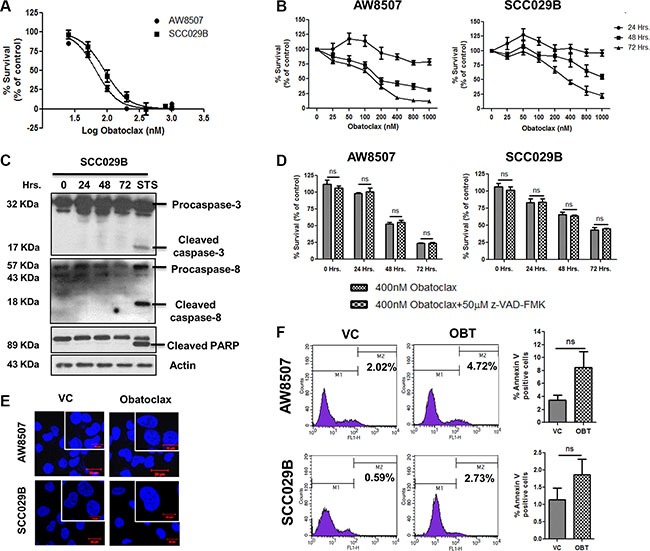
Obatoclax induces a caspase-independent cell death in OSCC cells (**A**) AW8507 and SCC029B cells were treated with increasing doses of Obatoclax for 72 hours and cell viability was assessed by MTT assay. (**B**) The cells were treated with increasing concentrations of Obatoclax for 24, 48 and 72 hours and cytotoxicity was assessed by SRB assay. Cell viability is expressed as percent survival with respect to vehicle control. (**C**) SCC029B cells were treated with 400 nM Obatoclax for 0, 24, 48 and 72 hours or 500 nM Staurosporine (STS) for 24 hours. Caspase and PARP cleavage were assessed by western blotting. (**D**) The cells were either treated with 400 nM Obatoclax alone or in combination with 50 μM z-VAD-FMK for 24, 48 and 72 hours and cell viability was determined by SRB assay. (**E**) The cells were treated with 400 nM Obatoclax for 48 hours and nuclei were stained with DAPI to analyze the nuclear morphology (Scale bar: 10 μm). (**F**) The cells were treated with 400 nM Obatoclax (OBT) for 72 hours or as vehicle control (VC) and the percentage of annexin V positive cells were measured by flow cytometry. Images are representative of three independent experiments. Data is represented as mean ± SEM of three independent experiments; ns indicate not significant.

Obatoclax induced a dose and time dependent decrease in the viability of OSCC cells as determined by MTT and SRB assay (Figure 2A and 2B). To investigate whether Obatoclax mediated cell death via the canonical caspase-dependent pathway, we exposed SCC029B cells to Obatoclax for 0, 24, 48 and 72 hours and assessed caspase activation by western blotting. Absence of caspase-3, caspase-8 and PARP cleavage upon Obatoclax treatment indicate a caspase-independent cell death (Figure 2C). This was further supported by the fact that OSCC cells exhibited no significant difference in the cell viability when treated with Obatoclax alone or in combination with a pan-caspase inhibitor Z-VAD-FMK (Figure 2D). Obatoclax treated cells showed no signs of nuclear fragmentation, characteristic of cells undergoing apoptotic cell death (Figure 2E). Analysis of cell death by flow cytometry revealed no significant increase in annexin V-positive cells in the Obatoclax treated population as compared to vehicle control cells (Figure 2F). All these observations point towards a caspase-independent, non-apoptotic form of cell death induced by Obatoclax in OSCC cells.

### Obatoclax induces autophagy in OSCC cells

**Figure 3:**
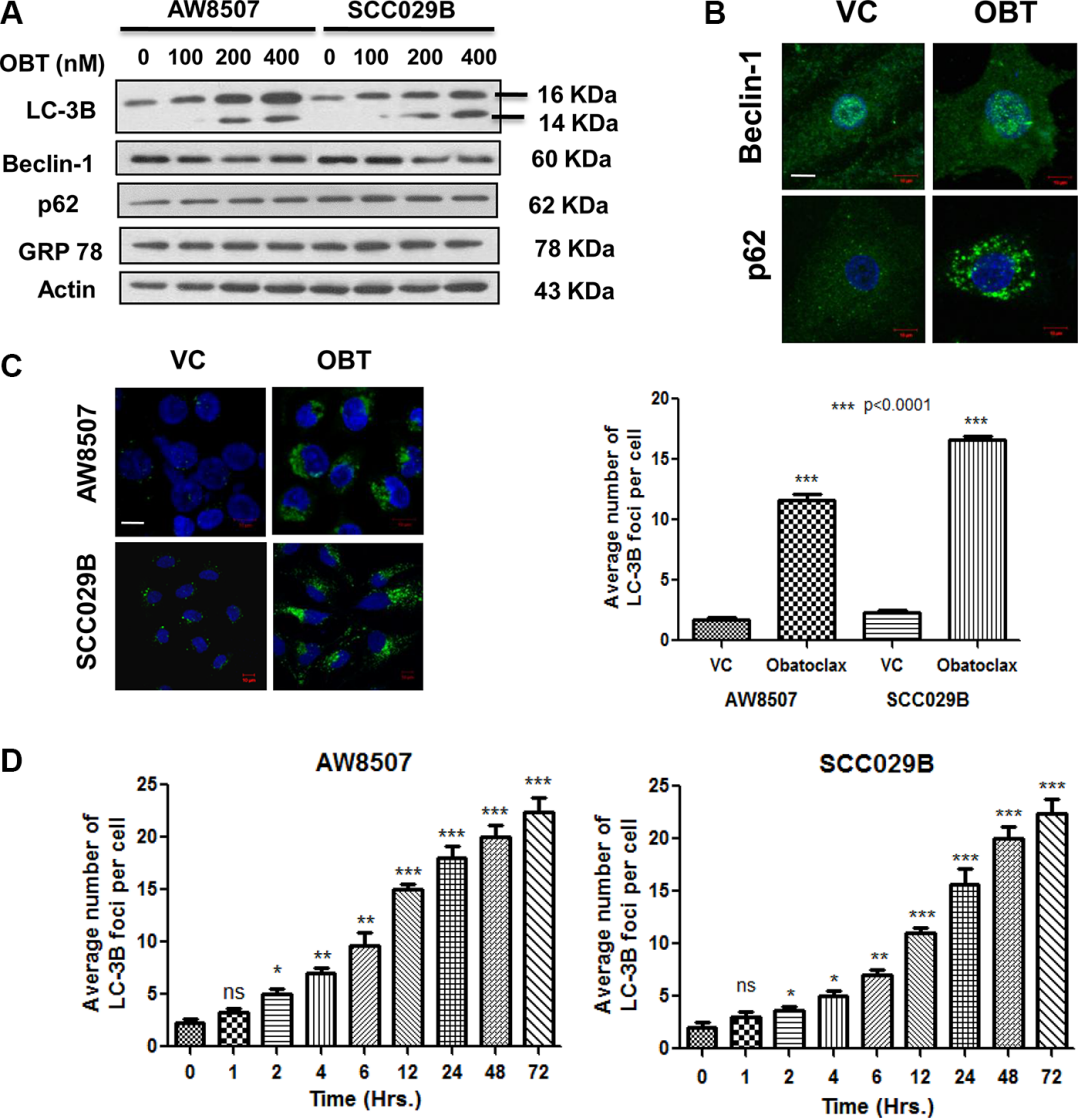
Obatoclax induces autophagy in OSCC cells (**A**) Autophagy induction in OSCC cells is evident from appearance of LC-3BII band when treated with increasing doses of Obatoclax for 24 hours. Beclin-1, p62 and GRP78 levels did not show significant alterations. (**B**) SCC029B cells were either treated as vehicle control (VC) or with 400 nM Obatoclax (OBT) for 24 hours and endogenous Beclin-1 and p62 were detected by immunofluorescence staining (Scale bar: 10 μm). (**C**) Quantitation of autophagy. AW8507 and SCC029B cells were either treated as vehicle control or with 400 nM Obatoclax for 24 hours and endogenous LC-3B was detected by immunostaining. The average number of LC-3B foci were scored in the Obatoclax treated and control cells (Scale bar: 10 μm). (**D**) A time course study of autophagy induced by Obatoclax. AW8507 and SCC029B cells were exposed to 400 nM Obatoclax for different time points and the average number of LC-3B foci per cell were detected as mentioned above. The data is represented as mean ± SEM of three independent experiments. The images are representative of three independent trials.

Endoplasmic Reticulum (ER) stress has been implicated in protection of human melanoma cells against the cell death induced by Obatoclax [44]. However, we observed no change in GRP78 (a marker of ER stress) expression, in response to Obatoclax treatment in OSCC cells. It has been shown that Obatoclax induces autophagy in a variety of cancer cells. Therefore, we next investigated whether Obatoclax induced autophagy in OSCC cells. Obatoclax induced autophagy in OSCC cells is evident from the appearance of LC-3BII band (which corresponds to LC-3B lipidation and its incorporation in the autophagosomal membranes). However, there was neither a significant change in Beclin-1 levels nor its subcellular localization. Notably, p62/SQSTM1 protein, a substrate of autophagy showed sustained levels and exhibited a punctate cytoplasmic appearance upon exposure to Obatoclax. The vehicle control cells in contrast, showed a diffuse cytoplasmic staining of p62 (Figure 3A and 3B). Autophagy induced by Obatoclax was further quantitated by immunofluorescence microscopy which depicted a significant (*p* < 0.0001) increase in the appearance of endogenous LC-3B foci in the Obatoclax treated versus control cells (Figure 3C). Autophagy induced by Obatoclax was also studied in a time course experiment which revealed appearance of numerous LC-3B foci in OSCC cells (Figure 3D).

### Obatoclax induced autophagy culminates into cell death

**Figure 4:**
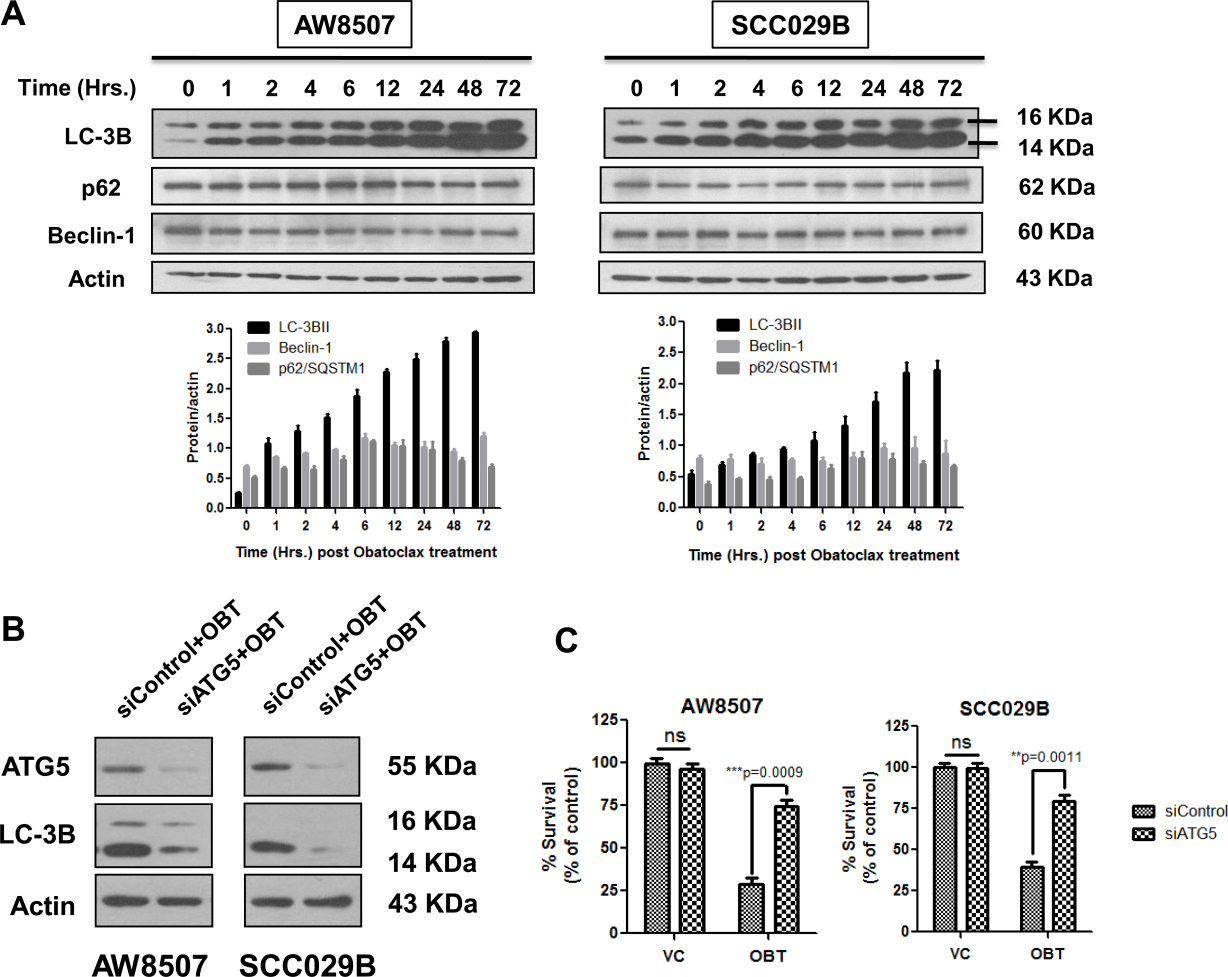
Obatoclax induced autophagy leads to cell death (**A**) A time course study of autophagy induced by Obatoclax revealed bulk accumulation of LC-3BII and sustained levels of p62/SQSTM1. The Beclin-1 levels however did not show significant changes. (**B**) AW8507 and SCC029B cells were transfected with either a control/scrambled siRNA (siControl) or siRNA against ATG5 (siATG5) and treated with 400 nM Obatoclax (OBT) for 24 hours. The levels of LC-3BII were detected by immunoblotting. (**C**) The siRNA transfected cells were treated with 400 nM Obatoclax for 72 hours and percent cell viability was determined by SRB assay. Western blots are representative of triplicate experiments. The data is represented as mean ± SEM of three independent experiments.

We then studied Obatoclax induced autophagy in a time course experiment by LC-3B and p62 immunoblotting. Consistent with the appearance of numerous LC-3B foci in the Obatoclax treated cells, we observed excessive accumulation of LC-3BII and sustained levels of p62 in a time dependent manner. All these observations are suggestive of a defective autophagy (Figure 4A). To address whether the Obatoclax induced autophagy is death inducing, we downregulated ATG5 by using siRNA and evaluated the Obatoclax-induced autophagy by LC-3BII immunoblotting. ATG5 knockdown significantly reduced the levels of LC-3BII in Obatoclax treated AW8507 and SCC029B cells (Figure 4B). Inhibition of autophagy by ATG5 downregulation significantly (*p* < 0.001) protected both the cell lines from Obatoclax-induced cell death (Figure 4C). These observations suggest that Obatoclax induced autophagy ultimately culminates into cell death.

### Obatoclax causes a disturbance in autophagic flux leading to a defective autophagy in OSCC cells

**Figure 5:**
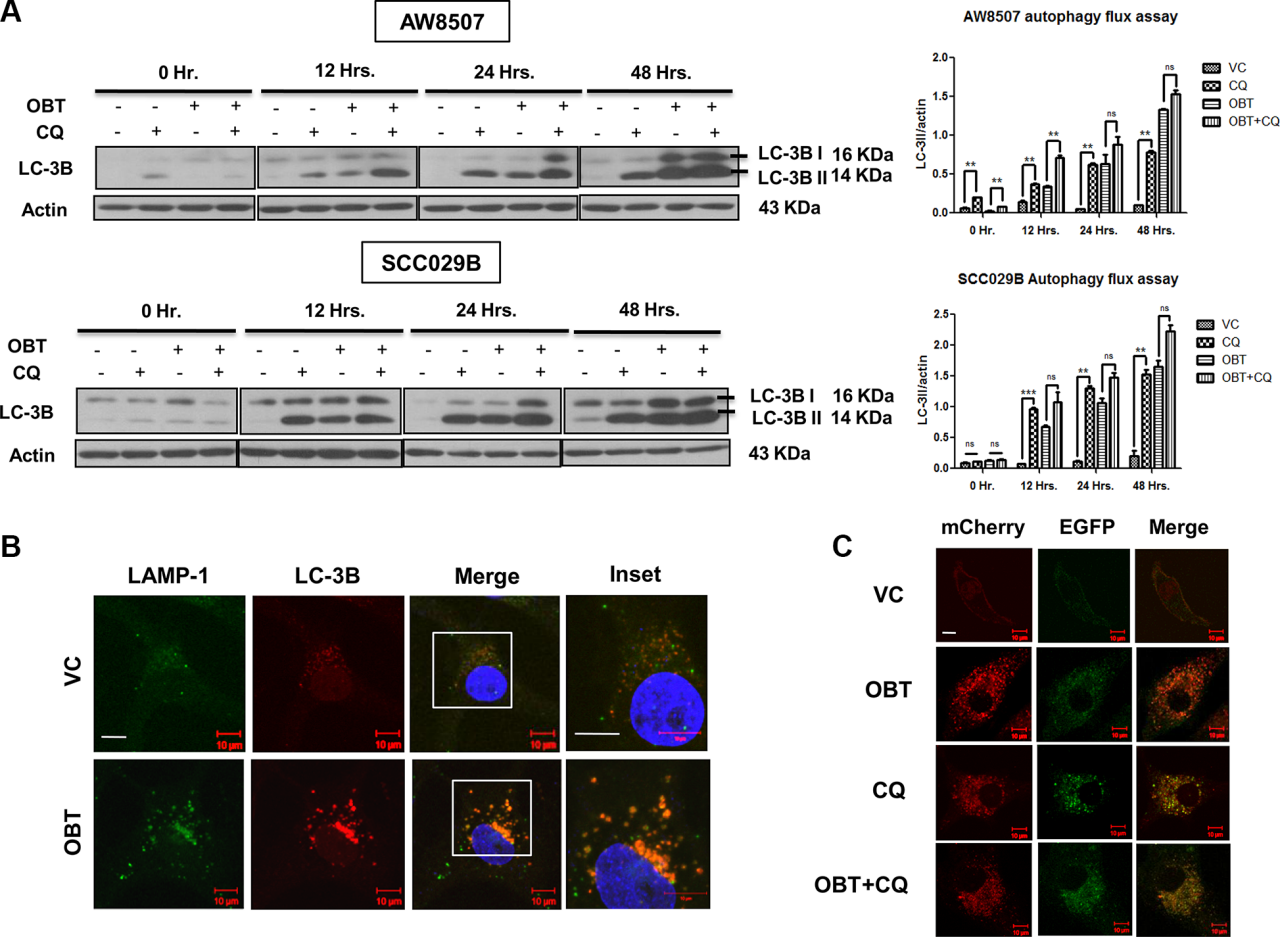
Obatoclax induces a defective autophagy in OSCC cells (**A**) Time course study of autophagy flux. AW8507 and SCC029B cells were treated with either 50 μM Chloroquine (CQ), 400 nM Obatoclax (OBT), a combination of both or as vehicle control (VC) for 0, 12, 24, 48 hours and autophagy induction was evaluated by LC-3B immunoblotting. The blots are representative of three independent experiments. The respective densitometric analysis graphs are represented as mean ± SEM of three independent experiments (**p < 0.01, ***p < 0.001). (**B**) Obatoclax induces fusion of autophagosomes with lysosomes. SCC029B cells were either treated with 400 nM Obatoclax for 48 hours or as vehicle control. Fusion of autophagosomes (LC-3B: Red) with lysosomes (LAMP-1: Green) appear as yellow-orange dots (due to overlap of red and green) (Scale bar: 10 μm). (**C**) SCC029B cells stably expressing pBABE-puro mCherry-EGFP-LC3B construct were either treated with 50 μM Chloroquine, 400 nM Obatoclax, a combination of both or as vehicle control for 48 hours and the cells were imaged using a fluorescence confocal microscope (Scale bar: 10 μm).

To further address whether the excessive accumulation of LC3BII (and the autophagosomes) in response to Obatoclax treatment are a result of their increased formation or reduced clearance of the autophagosomes, we studied the autophagy flux in OSCC cells. We exposed the cells to Chloroquine (50 μM), a lysosomotropic agent, which neutralizes the acidic interior of the lysosomes, thereby inhibiting the activity of lysosomal enzymes and blocking the degradation of the autolysosomal cargo. We observed that Obatoclax and Chloroquine individually induced LC-3BII levels to an equal extent. Whereas, a combination of Obatoclax and Chloroquine induced only a marginal increase (not significant) in LC-3BII levels as compared to either agents alone. These observations indicate that the increased LC-3BII levels in response to Obatoclax treatment are accounted by a block in the terminal degradative phase (Figure 5A) [45]. By immunofluorescence studies, we confirmed that Obatoclax treatment induces fusion of autophagosomes (LC-3B) with lysosomes (LAMP-1) to form autolysosomes (Figure 5B). To further dissect the autophagosome maturation process, we next investigated whether the autolysosomes are functionally competent to degrade the vesicular cargo by tandem fluorescent-tagged LC-3B based reporter [46]. SCC029B cells stably expressing a tandem mCherry/EGFP-LC-3B construct, exhibited yellow-orange (merge of mCherry red and EGFP green) fluorescence upon Obatoclax treatment, indicating functional incompetency of the autolysosomes (The GFP but not mCherry signal is sensitive to acidic compartment of lysosomes). We observed similar merged yellow-orange fluorescence signal of LC-3B when the cells were exposed to Chloroquine alone or a combination of Obatoclax and Chloroquine indicating a block at the degradation step (Figure 5C). All these observations together indicate that Obatoclax induced impaired autophagy due to a block in the terminal degradative phase.

### Obatoclax induced cell death in OSCC cells is mediated by necroptosis

**Figure 6:**
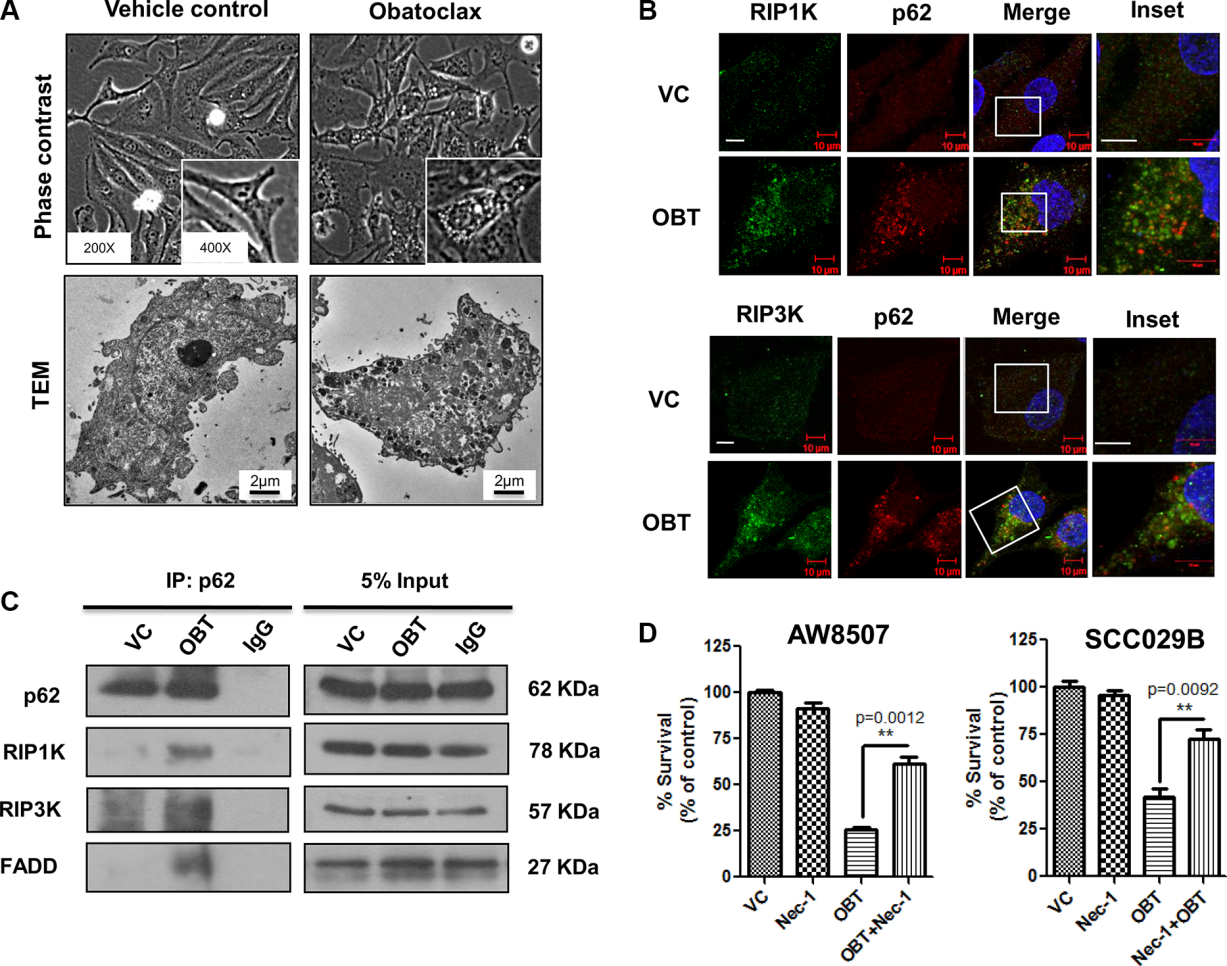
Obatoclax induces necroptosis in OSCC cells SCC029B cells were either treated as vehicle control (VC) or exposed to 400 nM Obatoclax (OBT) for 48 hours. (**A**) The morphology and ultrastructure of control and Obatoclax treated SCC029B cells. (**B**) A colocalization of p62 with RIP1K and RIP3K in Obatoclax treated SCC029B cells indicate assembly of necrosomal complexes (Scale bar: 10 μm). (**C**) The Obatoclax treated and vehicle control cell lysates were subjected to coimmunoprecipitation using p62 antibody. IgG: Isotype control antibody. (**D**) AW8507 and SCC029B cells were treated with 400 nM Obatoclax, 50 μM Necrostatin-1 (Nec-1) or a combination of both for 48 hours and cell viability was quantified by SRB assay. Data is represented as mean ± SEM of three independent experiments. Images are representative of three independent experiments.

Having shown that Obatoclax induced a caspase-independent, non-apoptotic cell death in OSCC cells, we looked for the cell death pathway linked to autophagy. It has been reported by several groups that defective autophagy in response to a variety of stress stimuli often leads to cell death via necroptosis [47, 48]. We therefore investigated whether necroptosis is involved in the cell death mediated by Obatoclax in OSCC cells. Obatoclax treated cells exhibited appearance of extensive cytoplasmic vacuolation, characteristic of cells under stress (Figure 6A). Recruitment of RIP1K and RIP3K at the necrosome is an important event during necroptosis. Here we demonstrate that Obatoclax induced the association of p62 with the components of the necrosome RIP1K, RIP3K, and FADD by colocalization and coimmunoprecipitation (Figure 6B and 6C). Moreover, treatment with Necrostatin-1 (Nec-1), a RIP1K inhibitor, partially protected the cells from Obatoclax-induced cell death (Figure 6D). Obatoclax thus appears to induce cell death in OSCC cells by necroptosis.

### Obatoclax induced extensive fragmentation of the mitochondrial network

**Figure 7:**
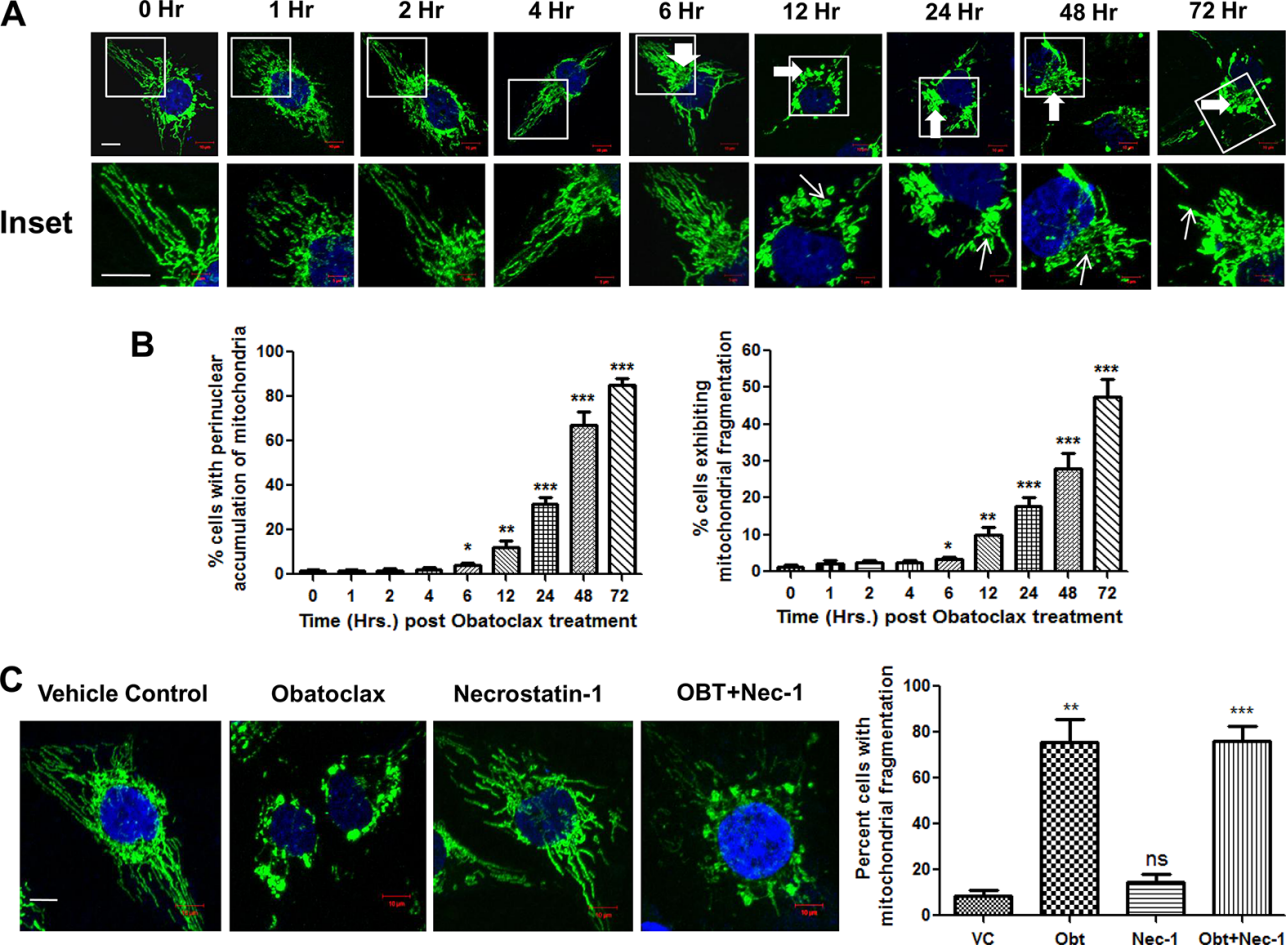
Obatoclax induces mitochondrial fragmentation in OSCC cells (**A**) SCC029B cells were treated with 400 nM Obatoclax for different time points followed by immunostaining for HSP60 and the nuclei were counterstained with DAPI. The cells were observed under a laser confocal microscope. The mitochondrial morphology is evident in the upper panels. The enlarged images are represented in the corresponding lower panels. (**B**) The percentage of cells showing perinuclear aggregation of mitochondria (indicated in the upper panels by thick white arrows) and those showing mitochondrial fragmentation (indicated by thin white arrows in the lower panels) were determined by counting 10 microscopic fields (each containing at least 10–15 cells) per time point. (**C**) SCC029B cells were treated with either 400 nM Obatoclax (OBT), 50 μM Necrostatin-1 (Nec-1), a combination of both or as vehicle control (VC) for 48 hours, immunostained for HSP60 and the percentage of cells exhibiting mitochondrial fragmentation was determined as described above. The data for all experiments is represented as mean ± SEM of three independent experiments (ns: not significant, *p < 0.05, **p < 0.01, ***p < 0.0001). The images are representative of three independent experiments (Scale bar: 10 μm).

Given the fact that MCL-1 is critical for mitochondrial homeostasis [22] and that putative MCL-1-specific inhibitors induce mitochondrial fragmentation [49], we speculated that Obatoclax could possibly deteriorate the organization of the mitochondrial network through MCL-1 inhibition. Obatoclax caused disruption of the mitochondrial network which involved extensive fragmentation, significant (*p* < 0.05) increase in mitochondrial fission and perinuclear aggregation in a time course study. The aberrations in the mitochondrial network initiated at about 12 hours post Obatoclax treatment and appeared much earlier than the phenotypic changes associated with the cell death (Figure 7A and 7B). Necroptosis is believed to execute cell death by inducing mitochondrial stress as one of the principal effector mechanism [50]. To investigate whether Obatoclax induced mitochondrial fragmentation occurs downstream of necroptosis, we treated SCC029B cells with Obatoclax in the presence or absence of RIP1K inhibitor Necrostatin-1 and assessed its effect on the mitochondrial network by HSP60 immunostaining. Mitochondrial fragmentation was evident even when RIP1K activity was inhibited, indicating that it occurs upstream to and independent of necroptosis (Figure 7C).

### Obatoclax induces mitochondrial oxidative stress and mitochondrial membrane depolarization in OSCC cells

**Figure 8:**
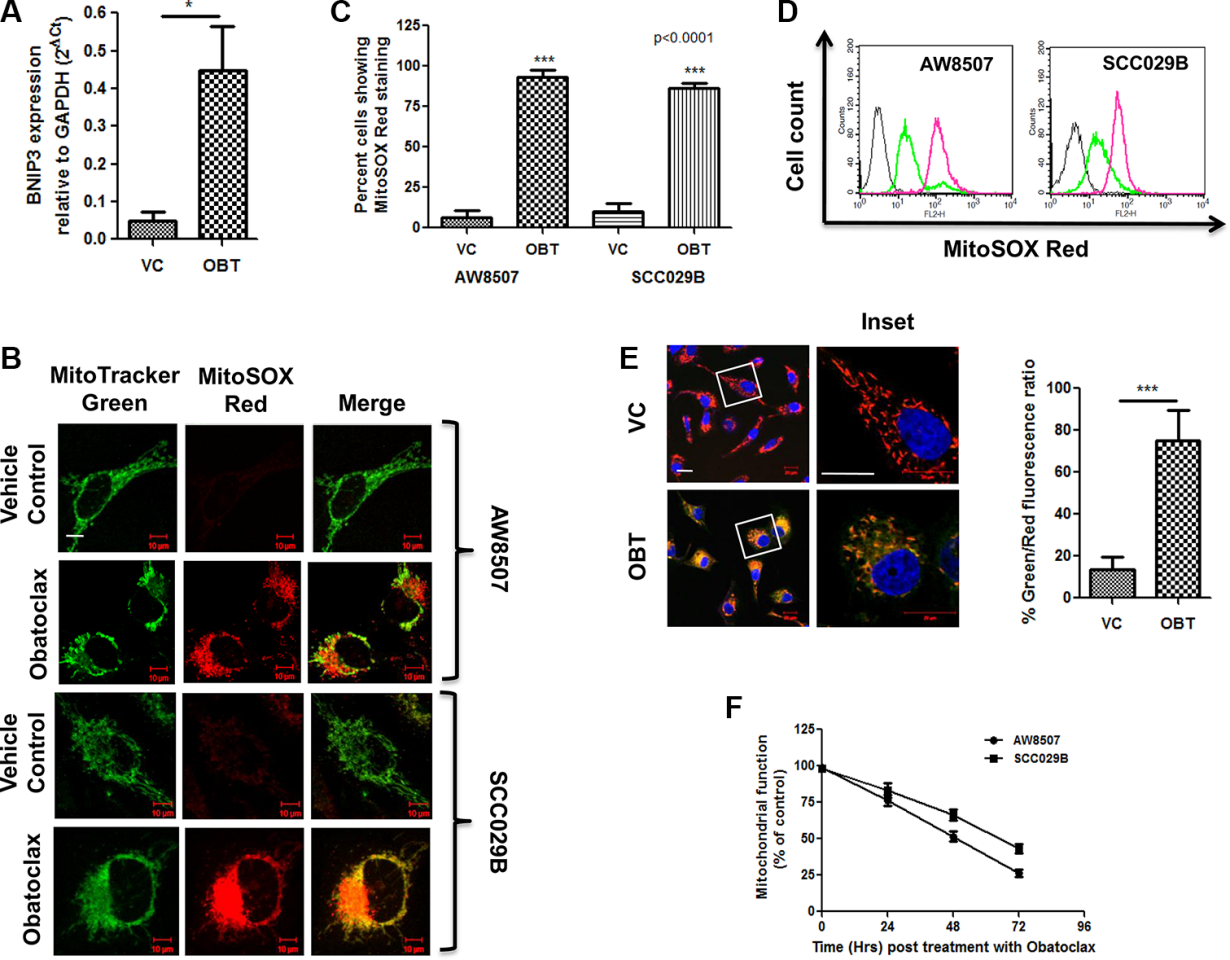
Obatoclax induces oxidative stress and membrane depolarization in the mitochondria of OSCC cells AW8507 and SCC029B cells were treated with 400 nM Obatoclax (OBT) for 48 hours or as vehicle control (VC). (**A**) The expression of BNIP3 was analyzed by real time PCR in SCC029B cells and expressed relative to GAPDH by 2^−ΔCt^ method. (**B**) AW8507 and SCC029B cells grown in confocal dishes were treated with Obatoclax or as vehicle control followed by staining with MitoTracker Green and MitoSOX Red. Images were acquired on a fluorescence confocal microscope (Scale bar: 10 μm). (**C**) The percentage of cells exhibiting MitoSOX Red fluorescence was determined by counting 10 different microscopic fields (each containing at least 10–15 cells) per condition. (**D**) Followed by drug treatment, the cells were stained with 5 μM MitoSOX Red dye and the amount of ROS accumulation in mitochondria was measured by flow cytometry on FL2 channel. The gray curve on extreme left indicate unstained cells, the green and pink curves represent vehicle control and Obatoclax treated cells respectively. The histogram plots are representative of three independent experiments. (**E**) The Obatoclax treated or vehicle control cells were stained with JC-1 dye and Hoechst dye. The percent Green/Red fluorescence intensity ratio was quantified by counting 10 different microscopic fields each containing at least 10 cells (Scale bar: 20 μm). Obatoclax induced mitochondrial membrane depolarization is indicated by a significant increase in the green/red fluorescence ratio of JC-1 dye as compared to the control cells. The red fluorescence was restricted to the mitochondria due to JC-1 aggregates and is indicative of a stable ΔΨm i.e. polarized mitochondria. The green fluorescence was diffused and corresponds to JC-1 monomers and indicates a drop in ΔΨm i.e. depolarized mitochondria. (**F**) Mitochondrial dysfunction measured by MTT assay. AW8507 and SCC029B cells were treated with 400 nM Obatoclax for 0, 24, 48 and 72 hours. The amount of reduced formazon dye is represented as percent mitochondrial function. Data is represented as mean ± SEM of three independent experiments.

BNIP3 (BCL-2-Nineteen kilodalton interacting protein 3) is a BH3 only protein of the BCL-2 family which plays a key role in impairment of the mitochondrial oxidative phosphorylation, reduction in the mitochondrial membrane potential (ΔΨm) and mitophagy under stressful conditions [51]. We therefore analyzed the expression of BNIP3 by real time PCR and observed that Obatoclax induced a significant increase (*p* < 0.05) in the expression of BNIP3 in SCC029B cells (Figure 8A). BNIP3 induction during stressful conditions leads to increased mitochondrial oxidative stress. We therefore investigated whether Obatoclax induced ROS (Reactive Oxygen Species) accumulation in mitochondria by using MitoSOX Red dye (MitoSOX Red dye specifically accumulates in mitochondria which upon oxidation by superoxide radicals exhibit red fluorescence). Live cell imaging experiment revealed that a significantly (*p* < 0.0001) higher percentage of Obatoclax treated cells exhibited MitoSOX Red fluorescence as compared to the control cells. This red fluorescence specifically localized to the perinuclear position consistent with the observation that mitochondria under stressful conditions exhibit perinuclear aggregation (Figure 8B and 8C). We also noticed a reduction in the fluorescence signal intensity of MitoTracker Green dye in the Obatoclax treated cells possibly because of a drop in ΔΨm. The increased ROS accumulation is also evident by flow cytometry, depicted by an increased proportion of MitoSOX Red positive cells upon Obatoclax treatment (Figure 8D). BNIP3 induction is also associated with a drop in ΔΨm. We therefore analyzed changes in ΔΨm by using JC-1 dye. As compared to the vehicle control, exposure of SCC029B cells to Obatoclax induced depolarization of the mitochondrial membrane reflected by a significant (*p* < 0.0001) increase in green to red fluorescence ratio of JC-1 dye. Increase in green fluorescence of JC-1 dye (due to JC-1 monomers) indicates a drop in ΔΨm whereas increased red fluorescence (due to JC-1 aggregates) corresponds to a stable ΔΨm and thus represents functional mitochondria (Figure 8E). Finally, we measured a time-dependent decrease in mitochondrial function upon Obatoclax treatment in OSCC cells by MTT assay (Figure 8F). All these observations point towards mitochondrial dysfunction upon Obatoclax treatment.

### MCL-1 downregulation induces mitochondrial stress in SCC029B cells

**Figure 9:**
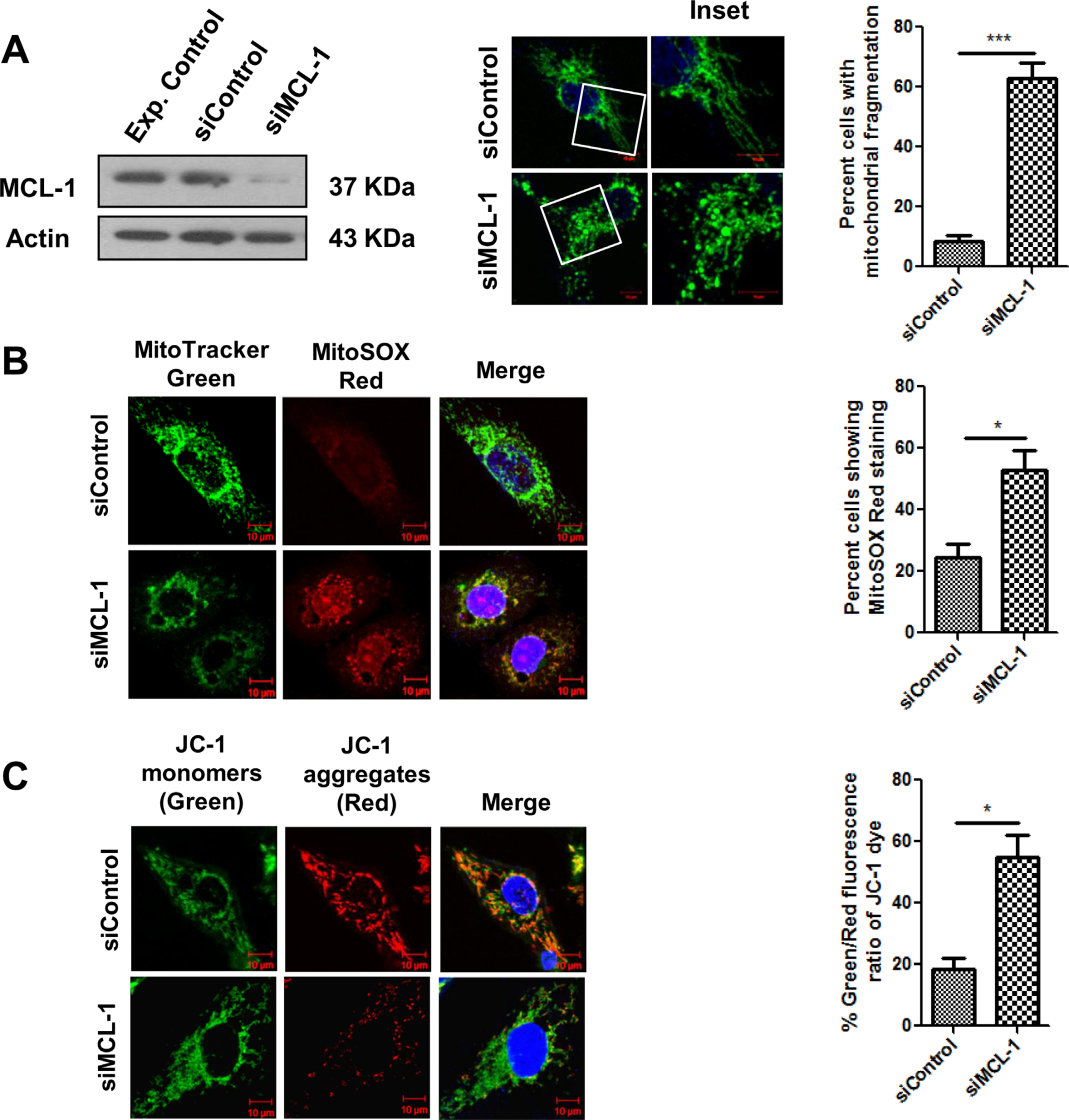
MCL-1 downregulation induces mitochondrial stress in SCC029B cells SCC029B cells were transfected with 50 nM control siRNA and 50 nM MCL-1 siRNA. 48 hours post transfection, cells were used for experimental analysis. (**A**) The cell lysates were subjected to western blotting for MCL-1 and β-actin. Alternatively, the siRNA transfected cells were immunostained for HSP60 and observed under a confocal microscope. The percentage of cells showing mitochondrial fragmentation was determined as described earlier. (**B**) The siRNA transfected cells were stained with MitoTracker green, MitoSOX Red and Hoechst dyes and observed under a confocal microscope. (**C**) The siRNA transfected cells were stained with JC-1 dye and analyzed for change in percent green to red fluorescence intensity ratio of JC-1 dye as discussed earlier. Data is represented as mean ± SEM of three independent experiments (Scale bar: 10 μm). Images are representative of three independent experiments.

To investigate the role of MCL-1 in mitochondrial homeostasis, we transiently knocked down MCL-1 by using siRNA in SCC029B cells. SCC029B cells as discussed earlier expressed relatively lower levels of BCL-2 and BCL-XL as compared to MCL-1. MCL-1 knockdown in these cells resulted in a significant (*p* = 0.0006) increase in mitochondrial fragmentation (Figure 9A). We also observed a significant (*p* < 0.05) increase in the MitoSOX Red staining in siMCL-1 transfected cells as compared to siControl cells (Figure 9B). Furthermore, MCL-1 downregulation resulted in a significant (*p* < 0.05) decrease in ΔΨm as compared to the control siRNA transfected cells (Figure 9C). All these observations indicate a potential role of MCL-1 in maintaining mitochondrial homeostasis in these cells.

### Ultrastructural changes induced by Obatoclax

**Figure 10:**
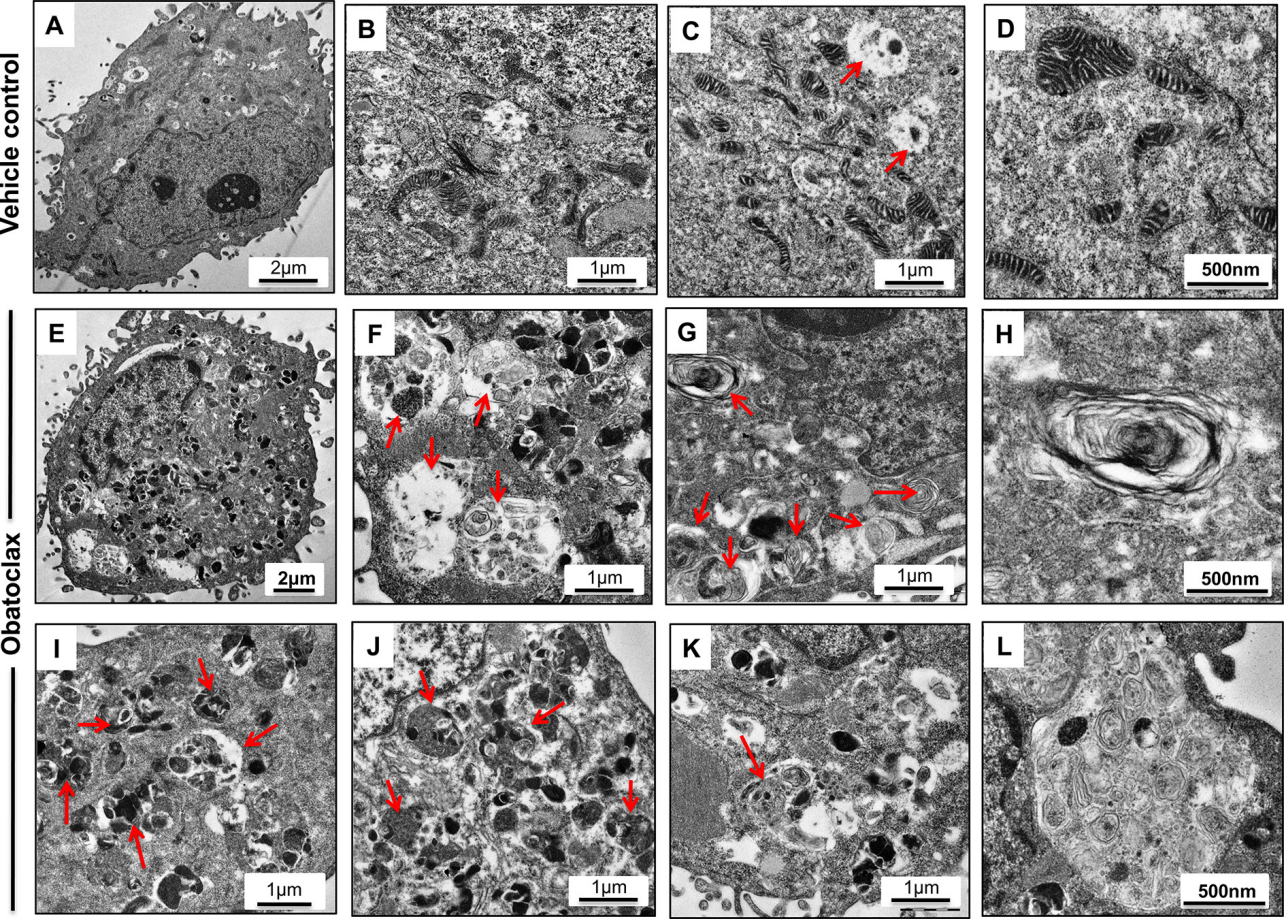
Ultrastructural changes induced by Obatoclax SCC029B cells when treated with 400 nM Obatoclax for 48 hours induced appearance of numerous autophagic vesicles (Red arrows) containing various cargos. Extensive cytoplasmic vacuolation was apparent with accumulation of electron dense proteinaceous inclusions. Intact mitochondria (those with distinct cristae and internal membranes) were not readily detectable (**E**–**L**). Vehicle control cells on the other hand exhibited significantly less cytoplasmic vacuolation and presence of numerous intact mitochondria with distinct cristae (**A**–**D**).

Obatoclax induced severe ultrastructural anomalies in OSCC cells. Numerous autophagic vesicles containing electron dense materials, protein inclusions and degenerating cellular organelles were readily visible throughout the cytoplasm. The autophagic vesicles at various stages of maturation ranging from initiating phagophore, autophagosomes, amphiosomes and autolysosomes were morphologically recognizable. Extensive cytoplasmic vacuolation was also apparent and intact mitochondria were not readily detectable. On the contrary, control cells exhibited numerous mitochondria (with intact inner and outer membranes and distinct cristae) and few cytoplasmic vacuoles (Figure 10).

### Obatoclax exhibits potent antitumor activity in xenograft mouse models

**Figure 11:**
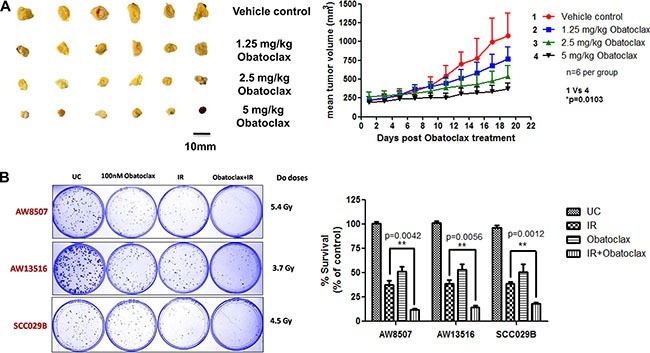
(**A**) *In vivo* efficacy of Obatoclax. BALB/C Nude mice bearing subcutaneous tumors derived from SCC029B cell line were administered a cumulative dose of the drug as indicated distributed evenly over a period of five consecutive days. Tumor volumes were measured for a period of 15–20 days post drug administration. The data is expressed as mean + SEM of the mean tumour volumes for each group (six animals per group). (**B**) Ionizing radiation treatment exhibits synergism when combined with Obatoclax. A combination of Obatoclax and ionizing radiation (IR) significantly (*p* < 0.05) inhibited the clonogenic potential of OSCC cells as compared to the either treatments alone.

Potent single agent antitumor activity of Obatoclax was observed against SCC029B cell line derived subcutaneous tumors in xenograft mouse model. We observed a significant (*p* < 0.05) reduction in the mean tumor volume without a significant decrease in the weight of the animals (Supplementary Figure S3). The effect was dose dependent with maximal activity observed at the cumulative dose of 5 mg/kg (Figure 11A).

### Obatoclax exhibits synergism with ionizing radiation to inhibit the clonogenic potential of OSCC cells

Consistent with our previous finding that MCL-1 is an important radioresistance related factor in OSCC cells [17, 18], we sought to test the potency of a combination of Obatoclax with ionizing radiation (IR). From the clonogenic assays, we observed that as compared to radiation alone, the combination treatment significantly (*p* < 0.05) inhibited the colony formation in the three OSCC cell lines (Figure 11B). Moreover, we observed a significant growth inhibition when the cells were exposed to a combination of IR and Obatoclax as opposed to radiation alone across a range of IR doses (2 Gy, 4 Gy, 6 Gy, 8 Gy) (Supplementary Figure S4). Obatoclax thus exhibit synergism with radiation treatment to inhibit the clonogenicity of OSCC cells.

## DISCUSSION

Aberrant expression of antiapoptotic BCL-2 family proteins, particularly MCL-1 has been reported in diverse human cancers including oral cancers [16, 52]. We have earlier demonstrated that the prosurvival MCL-1 protein contributes to therapy resistance and poor prognosis in oral cancers and thus may prove to be a potential therapeutic target in OSCC [15, 17–19]. Hence in the present study, we evaluated the efficacy of a BH3-mimetic pan-BCL-2 inhibitor Obatoclax against human oral cancer cell lines and also elucidated the mechanism of its action.

The four OSCC cell lines used in the present study exhibited high sensitivity to Obatoclax which correlated significantly to their MCL-1 expression. However, Obatoclax induced growth inhibition was not associated with a reduction in MCL-1 expression primarily because, Obatoclax is a BH3-mimetic which binds with a high affinity to the BH3-domain binding hydrophobic groove on MCL-1 protein and is not known to be associated with inhibition of its expression or mediate its degradation. This observation is in accordance with earlier studies reported in Cholangiocarcinoma cells [32, 53], Pancreatic cancer cells [31], KB carcinoma cells [26] and Breast cancer cells [42]. In contrast, studies by Yazbeck et al. demonstrated that sensitivity to Obatoclax has an inverse correlation with MCL-1 expression in HNSCC cells and is associated with a reduction in MCL-1 levels [43]. The discrepancy between these observations may be attributed to the cell type and context specific-dependence of the cells on MCL-1 for survival and the relative expression of other antiapoptotic members of the BCL-2 family. It is noteworthy that, all four OSCC cell lines expressed relatively higher levels of at least two of the three major antiapoptotic proteins (BCL-2, BCL-XL or MCL-1) which imply their dependence on multiple prosurvival proteins of the BCL-2 family for cell viability. Owing to their functional redundancy, tumor cells upregulate the expression of companion prosurvival BCL-2 family proteins in case, one of them is either inhibited or downregulated and therefore neutralization of all of them is necessary to execute apoptotic cascade [27, 28]. Hence the use of a pan-BCL-2 inhibitor like Obatoclax may prove to be an effective therapeutic strategy. In the present study, the growth inhibition induced by Obatoclax in OSCC cells was not associated with significant alterations in the expression of the BCL-2 family proteins except BIM and NOXA, whose levels were found to be reduced post Obatoclax treatment. Interestingly, NOXA and BIM downregulation partially protected H23 lung cancer cells from cell death induced due to MCL-1 knockdown [54] which probably explains why their expression is reduced upon Obatoclax mediated MCL-1 inhibition. Both BIM and NOXA are predicted to compete with Beclin-1 for interaction with MCL-1 and thereby regulate autophagy [42, 55, 56]. The regulation of expression of these BH3-only proapoptotic proteins therefore appears to be context and cell-type specific.

Recently, Wroblewski et al. has shown that Obatoclax induces unfolded protein response (UPR) in human melanoma cells which leads to MCL-1 upregulation, thereby preventing cell death [44]. UPR is characterized by increased GRP78 (Bip) expression, a principal chaperone in the endoplasmic reticulum (ER). However, in our studies, GRP78 expression remains unaltered in OSCC cells upon exposure to Obatoclax, indicating that Obatoclax may not induce ER stress in these cells. Obatoclax is reported to execute cell death via both caspase-dependent as well as caspase-independent pathways in a variety of cell types [31, 57]. In our studies, we observed that Obatoclax induced a caspase-independent, non-apoptotic cell death in OSCC cells.

Several studies have shown that Obatoclax potently induces autophagy in a variety of cancer cells [43, 57, 58]. Our present study also demonstrates autophagy induction in OSCC cells by Obatoclax which is evident from excessive accumulation of LC-3BII. BCL-2, BCL-XL and MCL-1 are known to bind Beclin-1 and thereby inhibit Beclin-1-mediated autophagy. Like BIM and NOXA, BH3 mimetics have been predicted to competitively disrupt this interaction and thereby mediate autophagy [36, 59]. However, we neither found significant alterations in Beclin-1 levels nor its altered subcellular localization pattern upon Obatoclax treatment in OSCC cells. McCoy et al. have demonstrated Beclin-1-indpependent autophagy induction by Obatoclax in non-small-cell lung carcinoma cells [57]. Blocking autophagy by ATG5 knockdown significantly protected the OSCC cells from Obatoclax-induced cell death, indicating that Obatoclax-induced autophagy leads to cell death and is not prosurvival. Obatoclax caused a disturbed autophagy flux which account for the bulk accumulation of LC-3BII and sustained levels of p62 protein (p62 is regarded as a substrate of autophagy which serves as a linker between polyubiquitinated cargo and LC-3BII on the autophagosomal membrane) in OSCC cells. A late-stage block in the autophagosome maturation process led to defective autophagy. More recently, Stamelos et al. have shown that Obatoclax accumulates in the lysosomes, mediates their alkalinization causing a block in the terminal degradative phase leading to a defective autophagy [60].

Having shown that Obatoclax induced a caspase-independent, non-apoptotic form of cell death in OSCC cells, we then investigated whether impaired autophagy culminates into cell death by necroptosis. Here, we demonstrate that Obatoclax induced the interaction of p62 with RIP1K, RIP3K and FADD, key components of the necrosome. Moreover, RIP1K inhibition by necrostatin-1 protected the OSCC cells from Obatoclax induced cell death. Our data thus suggests that Obatoclax mediates cell death in OSCC cells via autophagy-dependent necroptosis.

Our studies reveal that Obatoclax perturbed the normal tubular architecture of the mitochondria and induced extensive fragmentation of the mitochondrial network. This corresponds to increased mitochondrial fission and perinuclear aggregation. Initially it was believed that abrogation of the mitochondrial network, increased mitochondrial membrane permeability, mitochondrial ROS accumulation and mitochondrial dysfunction are one of the several effector mechanisms of Necroptosis [40, 61]. However, recent studies argue the involvement of mitochondrial fission and mitochondrial ROS during necroptotic cell death [62, 63]. Our data also suggests that mitochondrial fragmentation in response to Obatoclax treatment occurs independent of RIP1K activation and upstream of necroptosis.

Under stressful conditions, BNIP3 expression is induced which causes reduced energy output, increased accumulation of mitochondrial ROS and a drop in ΔΨm which mark the mitochondria as damaged and dysfunctional [51, 55]. On the same lines, we observed a significant increase in the expression of BNIP3 upon Obatoclax treatment. In accordance with the downstream effects of BNIP3 induction, we demonstrated a significant increase in the mitochondrial oxidative stress, compromised integrity of the mitochondrial membrane as indicated by a reduced ΔΨm (i.e. depolarized mitochondria) and a time-dependent decrease in mitochondrial function in these cells upon Obatoclax treatment. All the above characteristic events possibly mark the mitochondria as damaged and dysfunctional.

Besides its canonical prosurvival function, MCL-1 protein is critical for the organization and physiological functions of mitochondria [22]. Since MCL-1 is overexpressed in oral cancer cells and serve as an important prosurvival protein, we probed into its critical involvement in the maintenance of mitochondrial homeostasis in OSCC cells. In the present study, we evaluated and compared the effect of Obatoclax mediated MCL-1 inhibition (without a significant change in MCL-1 levels) with that of siRNA-mediated MCL-1 downregulation in context of mitochondrial architecture and physiology. MCL-1 downregulation in these cells led to extensive mitochondrial fragmentation, increased mitochondrial oxidative stress and a reduced mitochondrial membrane potential. These aberrations in the mitochondrial architecture and physiology were identical to those induced by Obatoclax treatment, thus highlighting the potential contribution of MCL-1 to normal mitochondrial organization and function. Perciavalle et al. provided evidence for contribution of MCL-1 to mitochondrial physiology. Apart from the antiapoptotic form of MCL-1 which localizes to the mitochondrial outer membrane, an amino-terminally truncated form of MCL-1 (contains intact carboxyl-terminus domain which harbors the BH3 domain binding hydrophobic groove where Obatoclax is predicted to bind) which localizes to the mitochondrial matrix plays a critical role in maintaining normal architecture, physiology, homeostasis, bioenergetics and fusion of the mitochondria [22]. Interestingly, putative MCL-1 specific BH3 mimetics such as BI97C1 and BI112D1 have also been shown to induce mitochondrial fragmentation, generation of ROS in mitochondria and inhibition of mitochondrial fusion independent of apoptosis and without affecting MCL-1 levels. In the same paper, the authors demonstrate extensive mitochondrial fragmentation upon siRNA mediated MCL-1 depletion in H23 lung cancer cells [49]. We therefore speculate that Obatoclax mediated MCL-1 antagonization may significantly contribute to the abolishment of mitochondrial structure and function.

We also demonstrate the single agent efficacy of Obatoclax in xenograft mouse model. Obatoclax exhibited dose dependent tumor regression in OSCC cell line derived subcutaneous tumors without detectable animal toxicity or weight loss. Moreover, consistent with our prior observation that MCL-1 is a critical radioresistance related factor in OSCC cells [18], Obatoclax exhibited a significant clonogenic inhibition in combination with ionizing radiation as opposed to the either treatments alone. The radiosensitizing effect of Obatoclax observed in the present study suggests that oral cancer patients may benefit from the combined therapeutic regimens including Obatoclax.

**Figure 12:**
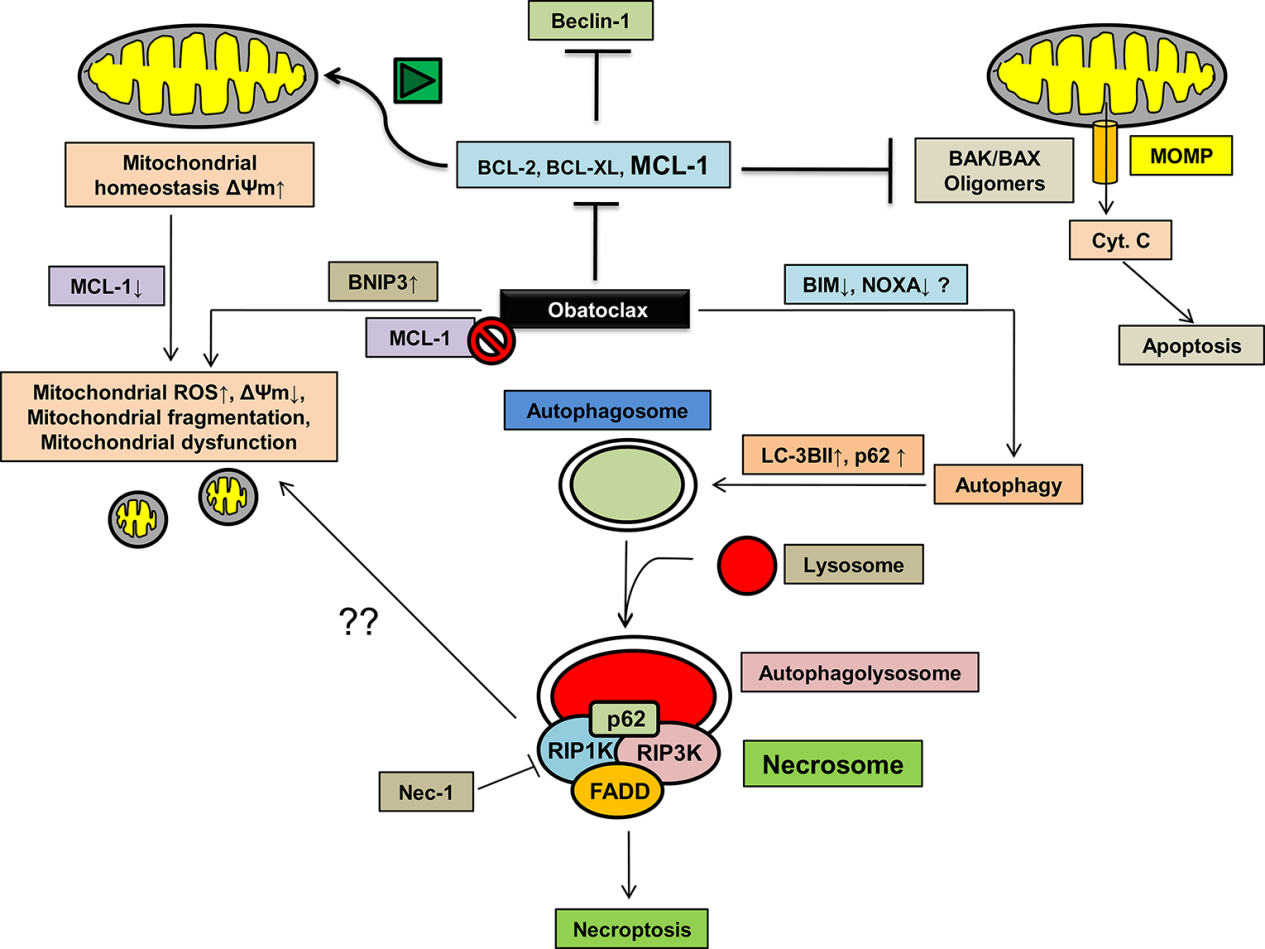
The proposed mode of Obatoclax action Obatoclax potently induces autophagy in OSCC cells. However, a late-stage block in the degradation step of autophagy leads to assembly of key proteins of the necrosome such as RIP1K, RIP3K and FADD at the autophagosomes along with p62/SQSTM1 in a complex called necrosome. Necroptosis is associated with extensive mitochondrial fragmentation, an induction of BNIP3 expression which potentiates ROS accumulation in the mitochondria, reduced mitochondrial membrane potential leading to mitochondrial dysfunction. Alternatively, whether or not MCL-1 inhibition mediated by Obatoclax at least partly contributes to the impaired mitochondrial homeostasis needs to be evaluated.

In summary, our studies indicate that Obatoclax which targets the prosurvival members of the BCL-2 family, specifically MCL-1, induces autophagy in OSCC cells leading to a caspase-independent, nonapoptotic form of cell death called necroptosis. These events are associated with extensive mitochondrial stress and dysfunction, are upstream to necroptosis and primarily attributed to Obatoclax mediated MCL-1 antagonization (Figure 12).

Although surgery remains the primary treatment modality for oral cancers, a multimodal approach including postoperative radiotherapy or chemotherapy (5-fluorouracil, platinum based drugs) is administered for advanced-stage disease [64]. However, we and others have reported the association of MCL-1 overexpression with resistance to radiation and cisplatin in oral cancers [18, 19]. Our studies also provide evidence for a synergistic combination of ionizing radiation and Obatoclax for the treatment of oral cancers. Although phase I clinical trials have demonstrated robust single-agent activity of Obatoclax in patients with advanced CLL [33] and small cell lung cancer [65], these were associated with neurotoxicity. Phase II clinical trials however, have not shown significant efficacy and were restricted by dose-limiting toxicity which includes thrombocytopenia, anemia, neutropenia, fatigue and ataxia [35]. Nevertheless, rational combination of treatments, systematic planning of therapeutic regimens and evaluation of molecular determinants of therapeutic outcomes may enhance the potency of Obatoclax.

We thus propose its potential application in the clinics for the better management of oral cancers.

## MATERIALS AND METHODS

### Cell culture

Four human oral cell lines derived from different oral subsites were used in the study. DOK (Dysplastic oral keratinocyte) derived from tongue epithelium [66] was cultured in DMEM (Gibco, USA) supplemented with 5 μg/ml hydrocortisone and 10% fetal bovine serum (Gibco). AW13516 and AW8507 cell lines were derived from poorly differentiated squamous cell carcinoma and epidermoid carcinoma of the tongue respectively [67]. UPCI:SCC029B cell line was derived from poorly differentiated squamous cell carcinoma of buccal mucosa [68]. AW8507, AW13516 and SCC029B cells were cultured in IMDM (Gibco) containing 10% fetal bovine serum. All the cell lines were authenticated by short tandem repeat (STR) profiling of 21 markers.

### Reagents and antibodies

#### Inhibitors, reagents and constructs

Obatoclax Mesylate (GX15-070) (Selleck chemicals, Texas, USA) was resuspended in DMSO to prepare a stock solution of 20 mM, aliquoted and stored at −20°C. It was brought to the desired working concentration by appropriately diluting in culture medium. 0.001% DMSO was used as vehicle control across all experiments. The pan-caspase inhibitor Z-VAD-FMK (Abcam, Cambridge, UK), Chloroquine and Necrostatin-1 (Calbiochem, USA) were used at 50 μM concentration. MTT reagent was dissolved in phosphate buffered saline (PBS) to a final concentration of 5 mg/ml. Changes in the mitochondrial network architecture were studied by using MitoTracker Green, MitoTracker Red CMXRos and MitoSOX Red dyes (Molecular probes, Invitrogen, USA). DAPI (4′,6-diamidino-2-phenylindole) or Hoechst 33342 (BD Biosciences, New Jersey, USA) were used to visualize nuclei. All standard chemicals were purchased from Sigma unless otherwise indicated. pBABE-puro mCherry-EGFP-LC3B construct was a gift from Jayanta Debnath (Addgene plasmid #22418). Control/Scrambled siRNA, siGLO, siRNA against human MCL-1 and ATG5 were obtained from Dharmacon (CO, USA). siRNA transfections were performed using Lipofectamine 2000 reagent (Invitrogen) according to the manufacturer's instructions.

### Antibodies

Primary antibodies against MCL-1 (sc-20679, sc-819), BCL-2 (sc-492), BAK (sc-832), NOXA (sc-26917), BID (sc-6538), Beclin-1 (sc-11427) and Actin (sc-1616R) were obtained from Santa Cruz Biotechnology (Texas, USA). Antibodies for p62 (#5114), HSP60 (#12165), BIM (#2933), BCL-XL (#2764), ATG5 (#12994), RIP1K (#4926) and RIP3K (#13526) were obtained from Cell Signaling technologies (Massachusetts, USA). Antibodies for PUMA (ab9643) and LC-3B (ab51520) were purchased from Abcam, and BAX (A3533) antibody from Dako (Denmark). For immunofluorescence, Alexa fluor 488, and Alexa fluor 568 secondary antibodies were used (Molecular probes).

### Colony formation assays

Briefly, cells were seeded in a 6-well plate (Nunc, Denmark) in duplicate and allowed to grow overnight followed by exposure to a range of Obatoclax concentrations for 24 hours. After the treatment, the drug containing medium was replaced with fresh growth medium and the cells were allowed to grow for further 10 days till appearance of visible colonies. The colonies were fixed and stained with Crystal Violet (Hi-Media Laboratories, India) and scored manually.

### Clonogenic assays to assess radiosensitivities in combination with Obatoclax

Exponentially growing cells were seeded in 35 mm plates and allowed to grow for 24 hours. Next day, the cells were irradiated (γ-irradiation on a ^60^Co source) with their respective D_0_ doses (previously determined by standard clonogenic assays [18]). Immediately after irradiation, the cells were exposed to 100 nM Obatoclax for 24 hours. After treatment, the drug containing medium was replaced with fresh growth medium and the cells were allowed to grow for further 8–10 days until visible colonies appeared in the control plate. The colonies were stained and scored as described earlier.

### Protein extraction and western blotting

The cells were harvested and lysed in cell lysis buffer (50 mM Tris pH 7.4, 150 mM NaCl, 0.5% NP-40) containing protease inhibitors (Fermentas, Canada). The cell lysates were incubated on ice for 30 minutes followed by centrifugation and the supernatants were subjected to protein estimation by Bradford assay (BioRad, USA). Equal amounts of proteins were resolved on SDS-PAGE and electroblotted onto PVDF membranes (Pall Technologies, USA). The membranes were blocked in 5% bovine serum albumin (BSA) in tris buffered saline (TBS) for 1 hour and then incubated with primary antibodies overnight at 4°C. Next day, the membranes were washed and incubated with secondary antibody at room temperature for 1 hour followed by washing. The proteins were visualized using enhanced chemiluminiscence kit (GE Healthcare, UK). Densitometric analysis was performed using the image J software (NIH, Bethesda, MD). β-actin served as the loading control. Mitochondrial fractionation from the cells was performed by using Mitochondria isolation kit (#89874, Thermo Scientific, IL, USA) according to manufacturer's instructions.

### Coimmunoprecipitation

The cells were harvested and lysed in EBC lysis buffer (50 mM Tris pH 7.4, 150 mM NaCl, 1 mM EDTA, 0.5% NP-40) containing protease inhibitors followed by centrifugation. The supernatants were precleared with protein G-sepharose beads (GE, UK) and subjected to overnight incubation with appropriate antibody at 4°C on a rocking platform. Next day, these cell lysates were incubated with protein G-sepharose bead slurry for 3 hours. The immunoprecipitates were recovered by gentle centrifugation, boiled in laemmli buffer and subjected to immunoblotting.

### RNA extraction, cDNA synthesis and real time PCR

Total RNA was extracted from cells by using TRIZol reagent (Invitrogen) according to manufacturer's instructions. 500 ng of total RNA was converted to cDNA by High Capacity cDNA synthesis kit (Applied Biosystems). The primers used for BNIP3 and GAPDH were as reported earlier [69]. Real Time PCR was performed on ABI QuantStudio 12 K Flex Sequence detection system (Applied Biosystems) using SYBR Green mastermix (Applied Biosystems). The results were analyzed by relative quantitation and expressed as 2^−ΔCt^.

### Flow cytometry

#### Measurement of ROS accumulation in mitochondria

The mitochondrial ROS accumulation was assessed by staining the cells with 5 μM MitoSOX Red for 10 minutes at 37°C in dark followed by acquisition on FL-2 channel of FACS caliber flow cytometer (Becton Dickinson, USA) and data was analyzed on Cell Quest software (BD Biosciences).

### Annexin V-FITC staining

After the drug treatment, the cells were harvested by trypsinization, washed twice with PBS and resuspended in Annexin V binding buffer (BD Biosciences) and incubated with Annexin V-FITC antibody (BD Biosciences) in dark for 15 minutes at room temperature followed by acquisition on FL-1 channel of FACS caliber flow cytometer and data was analyzed on Cell Quest software.

### Immunofluorescence and confocal microscopy

For live cell imaging, cells were cultured in confocal dishes and stained with 100 nM MitoTracker Green, 5 μM MitoSOX Red or 10 μM JC-1 dyes. For immunofluorescence staining, cells growing on coverslips were fixed with 4% paraformaldehyde followed by permeabilization with 0.5% triton X-100. Blocking was done in 5% BSA for 1 hour. The cells were then incubated with primary antibodies for 1 hour at room temperature followed by detection with appropriate secondary antibodies with 1 hour incubation at room temperature in dark. Nuclei were counterstained with DAPI. The coverslips were mounted with Vecta-shield (Vector laboratories, UK) on glass slides, sealed with nail polish and observed under a laser confocal microscope (LSM 510 Metaconfocal, Zeiss, Germany).

### Cell viability assays

#### MTT assay

Cells growing in the exponential phase were harvested by trypsinization and 2000 cells per well were seeded in 96-well plates (Nunc) and allowed to grow overnight. Next day, the medium was removed and the cells were treated with indicated concentrations of Obatoclax for the specified time points. At the end of the treatment period, 20 μl MTT was added to each well and the plates were again incubated in CO_2_ incubator for 4 hours. The formazon crystals were dissolved by addition of DMSO and absorbance was recorded on a microplate reader (Spectrostar nano, BMG Labtech, Germany) at 540 nm with a reference wavelength of 690 nm.

### Sulforhodamine B (SRB) assay

After the specified time point of drug treatment, the cells in 96 well plates were fixed with 30% Trichloroacetic acid (TCA) for 1 hour at 4°C followed by washing with water. The plates were then air dried and stained with SRB dye (0.05% w/v) for 30 minutes at room temperature. Excess dye was washed off by repeated washing with 1% acetic acid (v/v) and the plates were again air dried. Finally, the protein-bound dye was solubilized by addition of 10 mM Tris (pH 10) and absorbance was recorded at 540 nm with a reference wavelength of 690 nm on a microplate reader.

### Assessment of mitochondrial membrane potential (ΔΨm)

To study the effect of Obatoclax on the mitochondrial membrane potential (ΔΨm), we employed JC-1 dye (eBiosciences, San Diego, CA, USA) which is a cationic cell permeable dye and selectively accumulates in mitochondria in a potential dependent manner. Mitochondrial membrane polarization leads to the reversible formation of J-aggregates which causes a shift in the fluorescence emission from 530 nm (corresponding to JC-1 monomers which emits green fluorescence) to 590 nm (corresponding to J-aggregates which emits red-orange fluorescence). Cells growing in confocal dishes were incubated with 10 μM JC-1 dye for 30 minutes. The cells were then washed with PBS, replenished with fresh medium and observed under a fluorescence microscope.

### Transmission electron microscopy

Briefly, the cells were harvested by trypsinization and fixed with 3% glutaraldehyde at 4°C for 3–4 hours followed by washing with 0.1 M sodium cacodylate buffer. The cell pellets were then fixed in Osmium tetroxide for 1 hour at 4°C in dark, subjected to dehydration by passing through different grades of alcohol and then mounted with Araldite resin. The ultrathin sections (∼60–70 nm) were mounted on formvar coated copper grids. These sections were stained with uranyl acetate solution and counterstained with lead citrate. Electron micrographs were captured on a Jeol 100-CXII electron microscope (Jeol, UK) using Olympus camera and iTEM software.

### *In vivo* studies

#### Ethics statement

Investigation has been conducted in accordance with the ethical standards and according to the declaration of Helsinki and national and international guidelines. The study has been approved by the Institutional Animal Ethics Committee (IAEC) of Tata Memorial Centre (TMC)-Advanced Centre for Treatment, Research and Education in Cancer (ACTREC). To assess the *in vivo* antitumor activity of Obatoclax, we employed xenograft mouse model as described earlier [26]. Briefly, 6–8 weeks old female BALB/C nude mice were subcutaneously injected with 1 × 10^6^ SCC029B cells in 100 μl IMDM medium. The animals were then randomized into 4 groups, containing 6 animals each. Tumors were observed about 21 days post cell inoculation. Each group of animals were intravenously injected (through lateral tail vein) with different doses of Obatoclax (cumulative doses of 1.25 mg/kg, 2.5 mg/kg, 5 mg/kg and a vehicle control group) evenly distributed over a period of 5 days (i.e. 5 injections). The drug was formulated at the indicated concentrations in 9.6% PEG, 0.4% Tween 20 and 5% dextrose. The tumor volume measurements were done using a vernier caliper every alternate day by the formula: Length (mm) * [Width (mm)]^2^/2. For assessment of any drug associated toxicity, weight of the animals was monitored every alternate day.

### Statistical analysis

All the statistical analyses were performed using GraphPad Prism software (version 5.01). Two data sets in an experiment were compared by a two-tailed unpaired student's *t* test. The data is represented as mean ± Standard error mean (SEM). The difference between mean was considered significant when *p* < 0.05.

## SUPPLEMENTARY FIGURES


