# Supplementary material for: BH3 mimetic Obatoclax (GX15-070) mediates mitochondrial stress predominantly via MCL-1 inhibition and induces autophagy-dependent necroptosis in human oral cancer cells
Source: Oncotarget. 2016 Aug 5;8(36):60060–79. doi: 10.18632/oncotarget.11085 (PMC5601122; doi:10.18632/oncotarget.11085)
Supplement: Supplementary file 1 [file oncotarget-08-60060-s001.pdf]

BH3 mimetic Obatoclax (GX15-070) mediates mitochondrial stress predominantly via MCL-1 inhibition and induces autophagy-dependent necroptosis in human oral cancer cells

Supplementary Materials

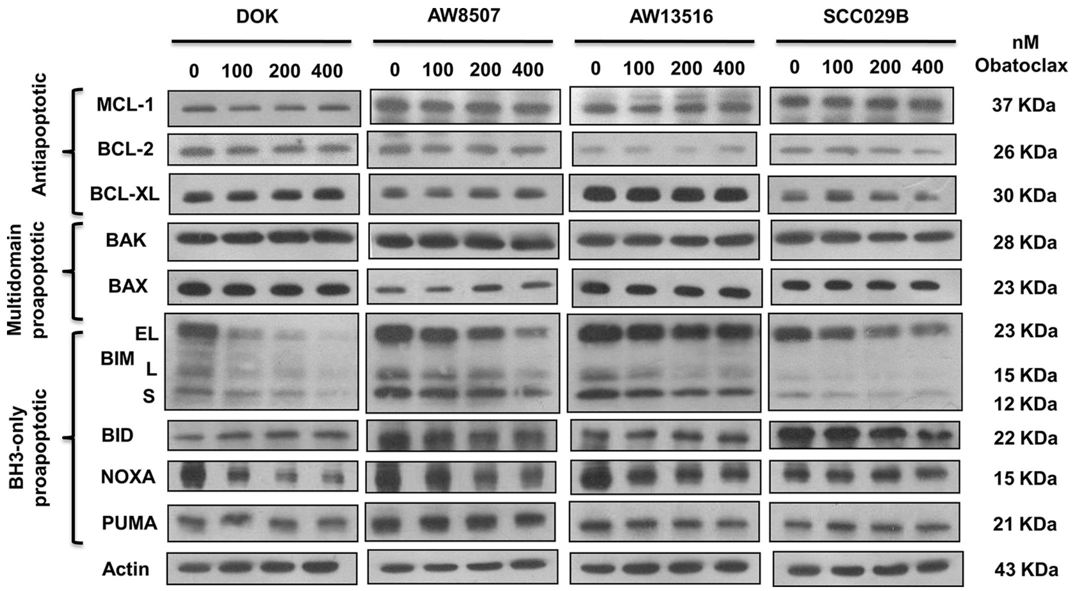

Supplementary Figure S1: Effect of Obatoclax on the expression of BCL-2 family proteins. The four OSCC cell lines were either treated with 100, 200 and 400 nM Obatoclax for 24 hours or as vehicle control. Equal amounts of whole cell lysates were subjected to immunoblotting for the indicated proteins. β-Actin served as the loading control. The blots are representatives of three independent experiments.

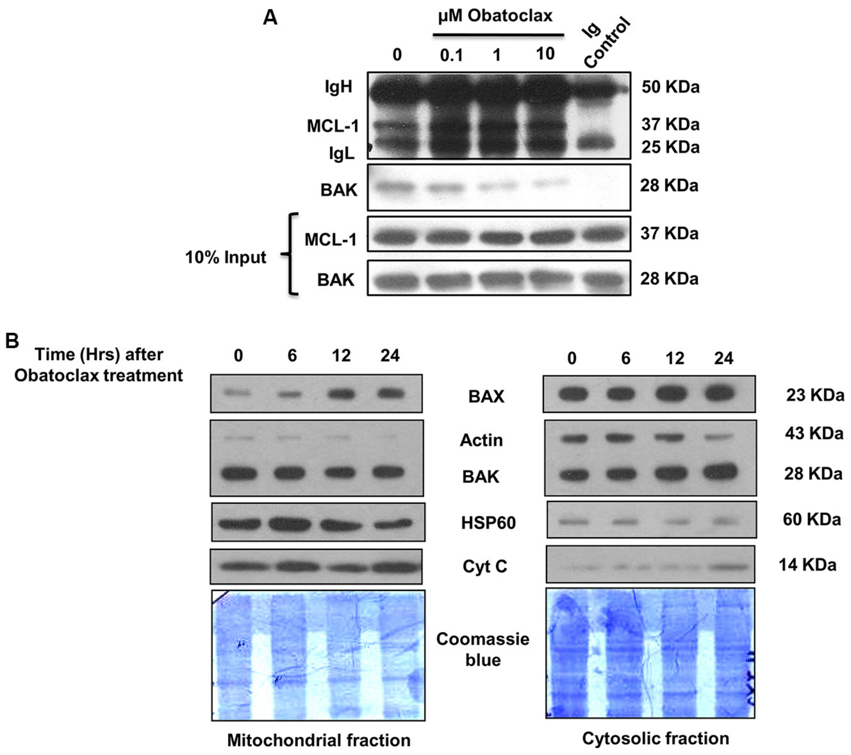

Supplementary Figure S2: (**A**) Obatoclax dissociates the interaction between MCL-1 and BAK. SCC029B cells were treated with 0, 0.1, 1 and 10 µM Obatoclax for 6 hours. The cell lysates were then subjected to immunoprecipitation with anti-MCL-1 antibody. The immunoprecipitates were probed with MCL-1 and BAK antibodies in a western blot. 10% input was included as a confirmation of presence of the proteins in the cell lysates used for immunoprecipitation reaction. (**B**) SCC029B cells were treated with 400 nM Obatoclax and harvested at different time points. The cells were fractionated into mitochondrial and cytosolic fractions. Equal amounts of mitochondrial and cytosolic fractions were analyzed for BAX, BAK and Cytochrome c Actin and HSP60 served as the loading controls for cytosolic and mitochondrial fractions respectively and to represent the purity of the preparations. BAX levels increased in the mitochondrial fraction in a time dependent manner which indicated increased BAX translocation to the mitochondria. BAK levels however remained unchanged.

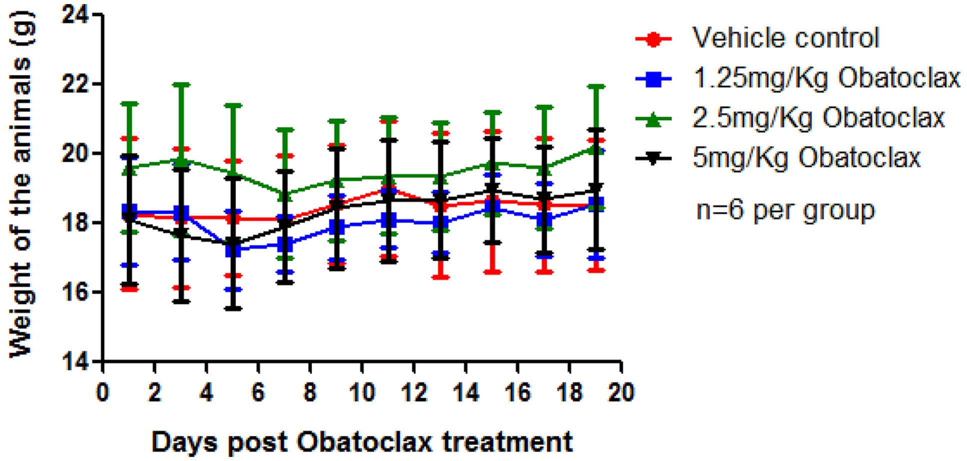

Supplementary Figure S3: Obatoclax does not exhibit toxicity in the animals. The weight of the animals was monitored every alternate day post Obatoclax administration upto about 18–20 days. Obatoclax did not exhibit a significant change in the weight of the animals in either a dose or time-dependent manner which is indicative of absence of toxicity in the animals.

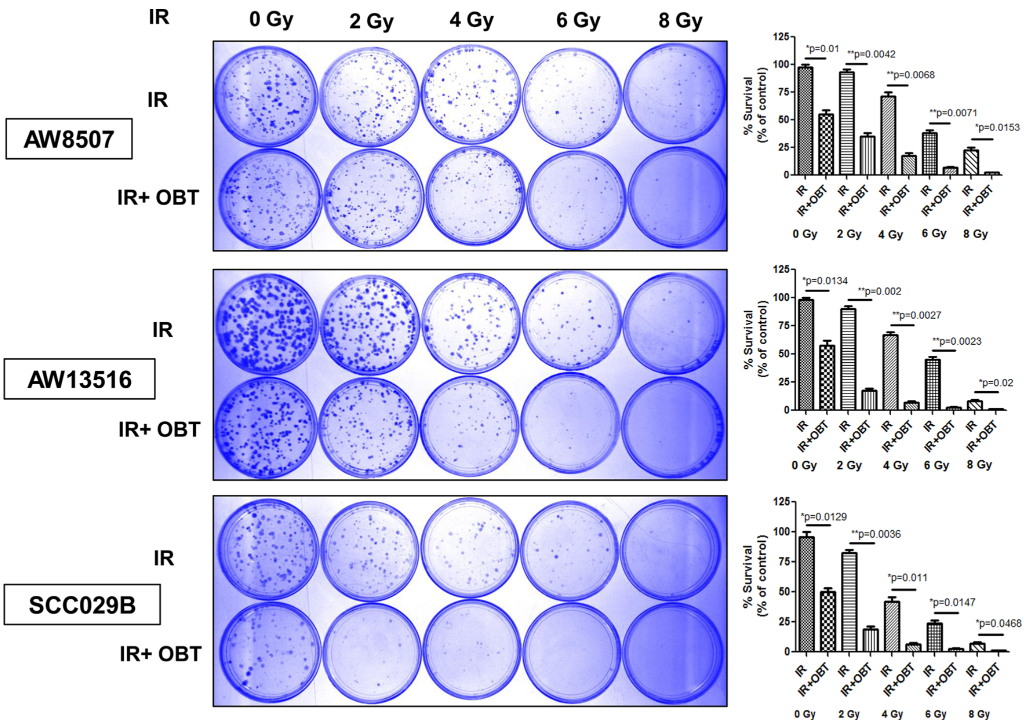

Supplementary Figure S4: Obatoclax exhibits synergism with ionizing radiation in a dose dependent manner. AW8507, AW13516 and SCC029B cells were treated with different doses of ionizing radiation (0, 2, 4, 6, 8 Gy) with or without 100 nM Obatoclax for 24 hours and the colony forming units were represented as percent viability. Data is represented as mean ± SEM of two independent experiments.
